# Test–retest reliability of Doppler ultrasound‐based leg blood flow assessments during exercise in patients with chronic obstructive pulmonary disease

**DOI:** 10.1113/EP092100

**Published:** 2024-09-02

**Authors:** Milan Mohammad, Jacob P. Hartmann, Amalie B. Andersen, Helene L. Hartmeyer, Ulrik W. Iepsen, Ronan M. G. Berg

**Affiliations:** ^1^ Centre for Physical Activity Research Copenhagen University Hospital – Rigshospitalet Copenhagen Denmark; ^2^ Department of Biomedical Sciences, Faculty of Health and Medical Sciences University of Copenhagen Copenhagen Denmark; ^3^ Department of Clinical Physiology and Nuclear Medicine Copenhagen University Hospital – Rigshospitalet Copenhagen Denmark; ^4^ Department of Anesthesiology and Intensive Care Copenhagen University Hospital Hvidovre Hospital Copenhagen Denmark; ^5^ Neurovascular Research Laboratory, Faculty of Life Sciences and Education University of South Wales Pontypridd UK

**Keywords:** cardiovascular physiology, methodology, physiolometrics, pulmonary disease, reproducibility, sonography, vascular capacitance

## Abstract

**Abstract:**

Doppler ultrasound may be used to assess leg blood flow (${\dot{Q}}_{\mathrm{leg}}$), but the reliability of this method remains unexplored in patients with chronic obstructive pulmonary disease (COPD), where between‐subject variability may be larger than healthy due to peripheral vascular changes. This study aimed to investigate the reliability of Doppler ultrasound in quantifying ${\dot{Q}}_{\mathrm{leg}}$ during single‐leg knee‐extensor exercise (KEE) in COPD patients compared with those obtained from healthy matched controls. In this case–control study, 16 participants with COPD were matched based on sex and age with 16 healthy controls. All participants underwent measurement of ${\dot{Q}}_{\mathrm{leg}}$ using Doppler ultrasound in a KEE set‐up at various intensities on two separate visits. Confounding factors on ${\dot{Q}}_{\mathrm{leg}}$ were controlled for, and the ultrasound scans were consistently performed by the same sonographer. During exercise, smallest real difference (SRD) ranged from 367 mL to 583 mL in COPD and 438 mL to 667 mL in the control group. The coefficient of variation (CV) ranged from 7.9% to 14.3% in COPD and 9.4% to 10.4% in the control group. The intraclass correlation coefficient ranged from 0.75 to 0.92 in COPD and 0.67 to 0.84 in the control group. CV was lower in the control group during exercise at 0 W, but apart from that, reliability was not different between groups during exercise. Doppler ultrasound showed nearly equal reliability when evaluating ${\dot{Q}}_{\mathrm{leg}}$ in COPD patients and healthy individuals with a CV below 15% during exercise for both groups.

**Highlights:**

- **What is the central question of this study?**
  What is the between‐day reliability of Doppler ultrasound when quantifying leg blood flow during single‐leg knee‐extensor exercise in COPD patients compared to healthy matched controls?
- **What is the main finding and its importance?**
  This study demonstrates a coefficient of variation ranging from 7.9 to 14.3% during single‐leg knee‐extensor exercise for between‐day reliability when applying Doppler ultrasound to assess leg blood flow in patients with COPD. Furthermore, it offers insights into the peripheral circulatory constraints in COPD, as evidenced by diminished leg blood flow. This study is the first of its kind to evaluate the reliability of Doppler ultrasound in the assessment of the peripheral circulation during exercise in COPD.

## 1 INTRODUCTION

Chronic obstructive pulmonary disease (COPD) stands as a complex respiratory disorder in which exercise intolerance is a defining characteristic, caused by structural and functional abnormalities in the peripheral musculature and vascular system (Maltais et al., 2014). Accordingly, patients with COPD exhibit a blunted regional blood flow response to exercise, as documented in the leg muscles during single‐leg knee‐extensor exercise (KEE) using different methods for leg blood flow (${\dot{Q}}_{\mathrm{leg}}$) measurement (Richardson et al., 2004; Brønstad et al., 2012; Hartmann et al., 2022; Iepsen et al., 2017). Impaired leg blood flow during exercise in COPD patients can potentially lead to reduced oxygen delivery and increased muscle fatigue as an important clinical feature in this clinical population.

Notable advances in the understanding of skeletal muscle perfusion and oxygen uptake kinetics in exercising humans have been achieved through the thermodilution technique (Andersen & Saltin, 1985) during both KEE and conventional cycle ergometry (Grassi et al., 1996; Poole et al., 1991; Richardson et al., 1993). Since then, thermodilution has served as the reference method for measuring blood flow during exercise, particularly in the femoral vasculature, but necessitates invasive catheterization and has also been reported to provide unreliable measurements when ${\dot{Q}}_{\mathrm{leg}}$ is low, such as in resting conditions (Gliemann et al., 2018). This prompted a shift toward Doppler ultrasound (Rådegran, 1997; Rådegran & Saltin, 1999; Walløe & Wesche, 1988; Wesche, 1986), which allows for non‐invasive beat‐to‐beat continuous concomitant vessel imaging (known as B‐mode imaging) and Doppler tracing (Barber et al., 1974). This is particularly beneficial when investigating physiological responses during exercise. However, when used to study ${\dot{Q}}_{\mathrm{leg}}$, Doppler ultrasound still poses several methodological challenges, notably difficulties in securing stable recordings during intense limb movement at higher exercise intensities, as well as obtaining the lowest possible insonation angle consistently. Moreover, the anatomical localization of the bifurcation of the femoral artery varies substantially between individuals (Seto et al., 2017), while prominent abdominal fat might narrow the scanning site, both factors which may affect the accessibility for insonation. Together, all these factors can contribute to both intra‐ and interobserver variability (Gill, 1985; Gliemann et al., 2018).

While previous studies have mostly focused on the validity and test–retest reliability of Doppler ultrasound for ${\dot{Q}}_{\mathrm{leg}}$ assessment in healthy individuals (Groot et al., 2022; Hartmann et al., 2023), the test–retest reliability between COPD patients and healthy individuals has not yet been investigated. The test–retest reliability of ${\dot{Q}}_{\mathrm{leg}}$ measurements using Doppler ultrasound may be suboptimal in the COPD population due to increased arterial stiffness and atherosclerotic plaques prevalent in COPD patients (Mills et al., 2008; Roeder et al., 2020; Zhang et al., 2022). Moreover, a potentially lower adherence to a given experimental KEE protocol, especially at higher intensities, can also contribute. Altogether, this complicates the interpretation of findings from previous studies, the identification of treatment effects in individual patients, and the design of future studies on COPD patients investigating ${\dot{Q}}_{\mathrm{leg}}$ using Doppler ultrasound‐based ${\dot{Q}}_{\mathrm{leg}}$ during KEE as a physiological outcome measure.

The objective of the present study was to 1) provide between‐day test–retest reliability estimates of Doppler ultrasound‐based ${\dot{Q}}_{\mathrm{leg}}$ at rest and during KEE in patients with COPD, and 2) to compare these estimates to those of age‐ and sex‐matched healthy controls.

## 2 METHODS

### 2.1 Ethical approval

The study was approved by the Regional Ethical Committee of the Capital Region of Denmark (file no. H‐23049997) and performed according to the most recent guidelines of the *Declaration of Helsinki*. All participants provided oral and written informed consent prior to enrolment. The study was registered on ClinicalTrials.gov (ID: NCT06135701).

### 2.2 Study design and setting

This case–control study is reported according to the Strengthening the Reporting of Observational Studies in Epidemiology (STROBE) Statement: Guidelines for Reporting Observational Studies (von Elm et al., 2008).

**FIGURE 1:**
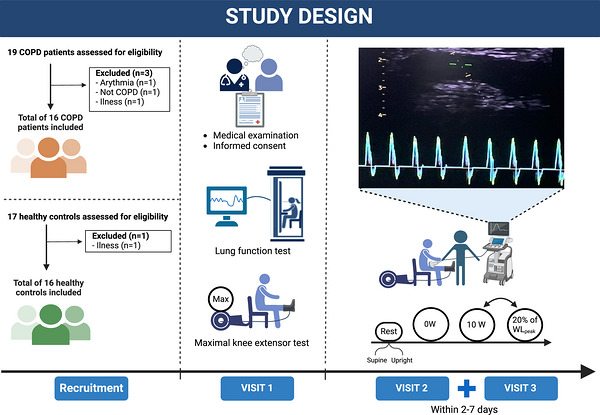
Study overview. A total of 16 COPD patients and 16 healthy controls underwent a single‐leg knee‐extensor exercise protocol with incremental workloads. This protocol was repeated within 2–7 day. *n*, number of patients; COPD, chronic obstructive pulmonary disease; WL_peak_, maximal workload attained during the maximal knee extensor test.

From 21 November 2023 to 12 January 2024, a total of 32 individuals were recruited to participate in the study at the Centre for Physical Activity Research, Rigshospitalet, Copenhagen, Denmark. We included 16 patients with COPD and 16 healthy controls. The study consisted of three visits on three different experimental days (Figure 1).

On visit 1, all participants underwent a brief medical examination, full lung function testing and an incremental KEE test, to determine peak workload (WL_peak_) measured in watts (W). On visits 2 and 3, participants underwent ultrasound examination of the common carotid artery on both sides to determine the carotid intima–media thickness (CIMT) as a surrogate marker of atherosclerosis. ${\dot{Q}}_{\mathrm{leg}}$ was measured by Doppler ultrasound in supine and seated positions in the resting condition and during KEE at different intensities. All participants were instructed to refrain from caffeine consumption (Umemura et al., 2006), alcohol ingestion (Carter et al., 2011), vaping and nicotine use (Chaumont et al., 2018) for at least 24 h before the Doppler ultrasound measurements on visits 2 and 3. They were furthermore instructed to refrain from vigorous exercise (Paterson et al., 2005) 48 h before the visits and to empty the bladder before tests.

Participants reported to visits 2 and 3, which were 2–7 days apart. For each participant, the experiments were performed at the same time of the day, but the time of day differed between participants. Furthermore, the experiments were performed in the same room with limited light exposure, controlled temperature, no music, and limited conversation. The Doppler ultrasound measurements were performed by the same experienced sonographer.

### 2.3 Participants

The inclusion criteria for COPD patients were (1) age from 45 to 80 years, (2) forced expiratory volume in 1 s (FEV_1_) to forced vital capacity (FVC) ratio (FEV_1_/FVC ratio) below 0.7, (3) a modified Medical Research Council score (mMRC) of 0–3, and (4) a resting arterial oxygenation >90% without supplemental oxygen. The COPD diagnosis was confirmed by a medical team of licensed physicians after clinical history and lung function test were assessed according to consensus criteria (Agustí et al., 2023; Cornelius, 2024). The inclusion criteria for healthy controls were (1) age from 45 to 80 years, (2) normal values of FEV_1_, FVC and FEV_1_/FVC ratio, (3) a mMRC of 0–3, (4) a resting arterial oxygenation >90%, (5) a normal single‐breath diffusion capacity for carbon monoxide corrected for haemoglobin (*D*_LCOc_).

Exclusion criteria for both COPD and healthy controls were known heart failure, ischaemic heart disease, cardiac arrhythmic disease, claudication, renal or liver dysfunction, malignant disease, pregnancy and symptoms of any disease within 2 weeks prior to the study. In COPD patients, participation in pulmonary rehabilitation within 6 months was also an exclusion criterion. For healthy controls, any kind of pulmonary disease was an exclusion criterion.

The cases (COPD patients) were individually matched on sex and age (±3 years) with controls (healthy individuals) in a 1:1 ratio.

### 2.4 Measurements

#### 2.4.1 Medical examination

During initial visit 1 (baseline), each study participant underwent a medical examination. This included auscultation of the heart and lungs, a concise medical history review, electrocardiogram, blood pressure, heart rate, and all current drug use to confirm the absence of any exclusion criteria conflicts. Height (m) and weight (kg) were measured, and body mass index (BMI, kg/m^2^) was calculated as weight/height^2^.

#### 2.4.2 Lung function testing

All lung function tests were conducted at the Centre for Physical Activity Research, Rigshospitalet, Copenhagen, Denmark by trained personnel following standardised protocols in line with international guidelines (Bhakta et al., 2023; Graham et al., 2019) as a part of the initial assessment. All tests were performed with Jaeger MasterScreen PFT pro system (CareFusion, Höchberg, Germany), and included dynamic spirometry, whole‐body plethysmography, and single‐breath uptake of carbon monoxide (CO). The following data were obtained: FEV_1_, FVC, FEV_1_/FVC ratio, total lung capacity (TLC), residual volume (RV), alveolar volume (*V*_A_) and *D*_LCOc_, as both absolute values and percentage of predicted according to standard reference equations (Stanojevic et al., 2022). Prior to measurements, patients' standing height (rounded to the nearest 1 mm), weight (rounded to the nearest 100 g), and haemoglobin (Hb) levels (rounded to the nearest 0.1 mmol/L) were obtained. Haemoglobin measurements were performed using capillary blood samples and analysed with the HemoCue device (Hb 201+; HemoCue AB, Ängelholm, Sweden).

#### 2.4.3 Maximal knee extensor test

The maximal workload (WL_peak_) of the quadriceps muscles was determined by 5 min of KEE on a customised single‐leg KEE chair at 6 W followed by incremental steps of 6 W/min until total exhaustion was reached as done in previous studies (Iepsen et al., 2017, 2021; Munch et al., 2018). Prior to the test, participants received verbal instructions and were familiarised to the KEE chair. Participants were instructed only to perform knee extension and not knee flexion. Proper form and technique were emphasised to isolate the quadriceps muscles and prevent compensatory movements. The assessment was conducted using the participant's right leg. WL_peak_ was calculated by noting the number of completed 6 W/min intervals. In cases where a participant was unable to complete the full 60 s of the final interval, the exact duration spent in the last interval was recorded. This time, measured in seconds, was then divided by 6 W to accurately determine the additional workload achieved in this final interval.

#### 2.4.4 Single‐leg knee‐extensor exercise

At arrival, all participants were encouraged to visit the toilet and empty their bladder before the tests. Before being placed in the KEE chair, supine Doppler ultrasound measurements were performed after a minimum 5 min of rest in the supine position. The participant was then placed on the KEE chair, where the measurements were performed in the seated resting condition, with the leg secured to the pedal. Subsequently, participants performed KEE, so ${\dot{Q}}_{\mathrm{leg}}$ could be measured at the following incremental workloads: 0 W, 20% of WL_peak_ and 10 W depending on whether their 20% of WL_peak_ values were lower or higher than 10 W, as it was an incremental order. The duration of each workload was approximately 2.5 min without any breaks before the next incremental workload as done in other studies (Hartmann et al., 2023; Iepsen et al., 2017, 2021; Munch et al., 2018).

#### 2.4.5 Doppler ultrasound

${\dot{Q}}_{\mathrm{leg}}$ was measured by the same sonographer using Doppler ultrasound (Logiq E9, GE Healthcare, Milwaukee, WI, USA) equipped with a linear probe (9 MHz), as previously described (Hartmann et al., 2023). Two ${\dot{Q}}_{\mathrm{leg}}$ measurements were performed after 2.5 min at each workload to ensure steady state. The sonographer ensured that the site of the ${\dot{Q}}_{\mathrm{leg}}$ measurements was below the inguinal ligament and 2–3 cm above the bifurcation of the artery. Moreover, the recordings were obtained at the lowest possible insonation angle and always below 60°. The sample volume was maximised according to the width of the vessel and kept clear of the walls. Each Doppler tracing was averaged over 15 s. 2D gain, sample volume and sweep speed were kept constant throughout the entire study. The end‐systolic diameter of the common femoral artery (CFA) was measured at rest from arterial B‐mode images with the transducer parallel to the vessel. This was used to calculate ${\dot{Q}}_{\mathrm{leg}}$ as:

$${\dot{Q}}_{\mathrm{leg}}=\mathrm{Time}-\mathrm{averaged}\;\mathrm{mean}\;\mathrm{velocity}\times \mathrm{cross}-\mathrm{sectional}\mathrm{area}.$$

where the cross‐sectional area was calculated by π × radius^2^ (Gill, 1985). The software from the Logiq E9 ultrasound machine automatically traced the Doppler waveform and provided mean velocities but also peak velocities and end‐diastolic velocities. For this study, mean velocity was used for quantifying Doppler velocities.

#### 2.4.6 Carotid intima–media thickness

B‐mode ultrasound (Logiq E9, GE Healthcare, Milwaukee, WI, USA) equipped with a linear probe (9 MHz) was used to scan the common carotid artery on both sides to determine the carotid intima–media thickness (CIMT) as a measure of atherosclerosis (Nezu et al., 2016; van den Oord et al., 2013). CIMT was obtained on both sides with two measurements on each side on each visit equalling a total of four measurements per visit that were averaged. The CIMT was measured on the posterior intimal layer 1–2 cm inferior to the common carotid bifurcation (Casella et al., 2008). CIMT is a marker of universal atherosclerosis which can influence vascular function (Bauer et al., 2012; Willeit et al., 2020). CIMT is therefore evaluated to demonstrate the atherosclerotic burden in both groups, which may affect reliability as increased CIMT is associated with greater arterial stiffness and reduced compliance, which impacts ${\dot{Q}}_{\mathrm{leg}}$ (Köseoğlu et al., 2016; Mohamed et al., 2023; Naqvi & Lee, 2014).

### 2.5 Outcome measures

Absolute between‐day reliability was assessed by an estimation of the smallest real difference (SRD), and the corresponding relative reliability estimates were assessed by coefficient of variation (CV) and intraclass correlation coefficient (ICC).

### 2.6 Potential sources of bias

The likelihood of misclassification of COPD was minimal. The COPD diagnosis was rigorously validated through comprehensive lung function tests, adhering to widely accepted consensus guidelines (Agustí et al., 2023; Cornelius, 2024; Stanojevic et al., 2022) and evaluated by a medical team of licensed physicians. All measurements were standardised and conducted consistently by the same team with the same equipment. Notably, all ultrasound examinations were performed by a single, experienced sonographer utilising the same ultrasound equipment, ensuring methodological consistency. However, the predominantly Caucasian composition of our study cohort limits the applicability of our results across diverse ethnic backgrounds.

### 2.7 Sample size

To detect a between‐group (COPD vs. control) change in ${\dot{Q}}_{\mathrm{leg}}$ from rest to exercise of 200 mL/min, with a power of 90% and an alpha‐level of 5%, at least 12 participants were needed in each group based on a previous study (Iepsen et al., 2017). However, to account for potential dropouts and unexpected events, 16 participants were included in each group.

### 2.8 Statistical methods

All statistical analyses were conducted using R statistical software version 4.3.3 (R Foundation for Statistical Computing, Vienna, Austria) within RStudio (version 1.4.1717). Normality of the data was assessed with visual inspection by histograms and quantile–quantile (QQ) plots.

Normally distributed variables are reported as mean (SD) and mean difference (95% confidence interval (95% CI): lower limit, upper limit); otherwise, non‐normally distributed data are reported as median [25th percentile–75th percentile]. Student's *t*‐test was used to detect differences in baseline characteristics between groups and Mann–Whitney *U*‐test was used when assumption of normality failed.

Data were analysed using linear mixed effects regression (lme4 package; version 1.1–35.3; Bates, Maechker, Bolker, & Walker, 2015). Exact model terms were bloodflow ∼ day + group × condition + (1 | ID), with random intercepts for unique participant ID to account for repeated measures. Model assumptions were assessed via visual inspection of fitted values versus residuals plots. Post‐hoc pairwise testing of the marginal means was performed to investigate potential difference during different conditions (supine, seated, 0 W, 10 W and 20% of WL_peak_).

For absolute reliability, we estimated SRD, which estimates the maximum difference between any two measurements on 95% of occasions when no real underlying difference is present, using a one‐way analysis of variance (ANOVA). SRD is expressed as an absolute value in the same unit as the outcome. Moreover, one‐way ANOVA was used to determine the standard deviation within participants (SDw), and SRD was calculated using the following formula (Vaz et al., 2013):

$$\mathrm{SRD}\;=\;(T-\mathrm{quantile}\;\mathrm{with}\;\mathrm{appropriate}\;\mathrm{degrees}\;\mathrm{of}\;\mathrm{freedom}\times \sqrt{2\times \mathrm{SD}w^{2}}$$

Relative reliability was assessed by CV and ICC as an additional measure. CV expresses the proportion of variance (%) caused by measurement error and is calculated as (Vaz et al., 2013):

$$\mathrm{CV}\;=\frac{\mathrm{SDw}}{\mathrm{Mean}\;\mathrm{of}\;\mathrm{measurements}}\;\times 100$$

Based on the distribution of mean estimates and residual variance from a linear mixed model, we simulated the distribution of the CV to obtain 95% confidence intervals for CV, following the method previously described (Liu, 2012). ICC was calculated using a two‐way mixed‐effects model with the absolute agreement and multiple measurements ICC (Koo & Li, 2016; Lee et al., 2012).

The R‐package *clintools* was used to calculate SRD, CV and ICC with 95% CI and *P*‐values for between group comparisons by bootstrapping using 1000 iterations in the *comparerel* function from the *clintools* package (Hartmann et al., 2023; Olsen et al., 2023). Statistical significance was accepted at α < 0.05 (two‐sided). Of note, when using the *comparerel* function, exact *P*‐values for between‐group differences in reliability metrics were not provided but only indicated as *P* < 0.05.

## 3 RESULTS

### 3.1 Participant characteristics

Participant characteristics are outlined in Table 1. The two groups differed in that the COPD group had more pack years, lower FEV_1_, FEV_1_/FVC ratio, *D*_LCOc_, higher RV, systolic blood pressure, a greater use of antihypertensive medications and a lower WL_peak_ compared to controls. The median time interval between visit 2 and 3 was 4 (2–7) days for both groups, with no missing data observed for any participants.

**TABLE 1:** Baseline characteristics.

Characteristic	COPD	Controls	*P*
*n*	16	16	—
Female/Male	8/8	8/8	—
Age (years)	64.31 (5.64)	63.19 (5.04)	0.55
Height (cm)	172.60 (10.77)	170.45 (9.17)	0.54
Weight (kg)	81.38 (14.95)	74.03 (7.21)	0.09
BMI (kg/m^2^)	27.29 (4.58)	25.49 (1.68)	0.16
BSA (m^2^)	1.95 (0.21)	1.86 (0.14)	0.17
Pack years	38.70 (15.20)	5.38 (8.20)^a^	<0.001
Current smokers (*n*)	0	0	—
Former smokers (*n*)	15	6^a^	0.002
Medication use (*n*)			
Statin	5	3	0.68
Anti‐hypertensives	9	1^a^	0.01
Metformin	3	1	0.59
Systolic blood pressure (mmHg)	140 (14.59)	127 (15.88)^a^	0.02
Diastolic blood pressure (mmHg)	86.19 (9.60)	82.38 (10.72)	0.30
FEV_1_ (l)	1.98 (0.87)	3.22 (0.63)^a^	<0.001
FEV_1_ (% predicted)	65.63 (18.37)	108.31 (13.05)^a^	<0.001
FVC (l)	3.72 (1.38)	4.18 (0.81)	0.26
FVC (% predicted)	96.13 (25.33)	108.44 (12.29)	0.09
FEV_1_/FVC ratio	52.59 (8.91)	78.27 (5.21)^a^	<0.001
FEV_1_/FVC ratio (% predicted)	67.63 (11.35)	99.81 (6.91)^a^	<0.001
RV (l)	3.17 (0.89)	2.15 (0.35)^a^	<0.001
RV (% predicted)	140.56 (44.79)	97.84 (10.68)^a^	<0.001
TLC (l)	6.83 (1.55)	6.18 (1.02)	0.17
TLC (% predicted)	109.19 (17.90)	102.92 (11.50)	0.25
*D*_LCOc_ (mmol/min/kPa)	6.17 (2.57)	8.51 (1.54)^a^	<0.001
*D*_LCOc_ (% predicted)	70.13 (21.68)	99.08 (10.27)^a^	<0.001
Haemoglobin	9.23 (0.74)	8.88 (0.71)	0.52
Single‐leg watt max (W)	31.83 (18.03)	47.42 (14.40)*	0.01
20% of watt max (W)	6.37 (3.61)	9.48 (2.88)*	0.01
Breathlessness at watt max (1–5)	2 [1–4]	2 [1–4]	0.90
Muscle soreness at watt max (1–5)	4 [3–5]	4 [3–5]	0.80
Days between Doppler measurements	4 [2–7]	4 [2–7]	0.60
Right average CIMT (cm)	0.77 (0.17)	0.70 (0.14)	0.22
Left average CIMT (cm)	0.74 (0.18)	0.73 (0.11)	0.76
Combined average CIMT (cm)	0.76 (0.18)	0.72 (0.13)	0.31

*Note*: Data are in means (SD) or medians [IQR].

^a^ Different from COPD group (*P* < 0.05). Abbreviations: BMI, body mass index; BSA, body surface area; CIMT, carotid intima–media thickness; *D*_LCOc_, pulmonary diffusing capacity for carbon monoxide corrected for haemoglobin; FVC, forced vital capacity; FEV_1_, forced expiratory volume in 1 s; *n*, number of patients; RV, residual volume; TLC, total lung capacity.

### 3.2 Leg blood flow

**FIGURE 2:**
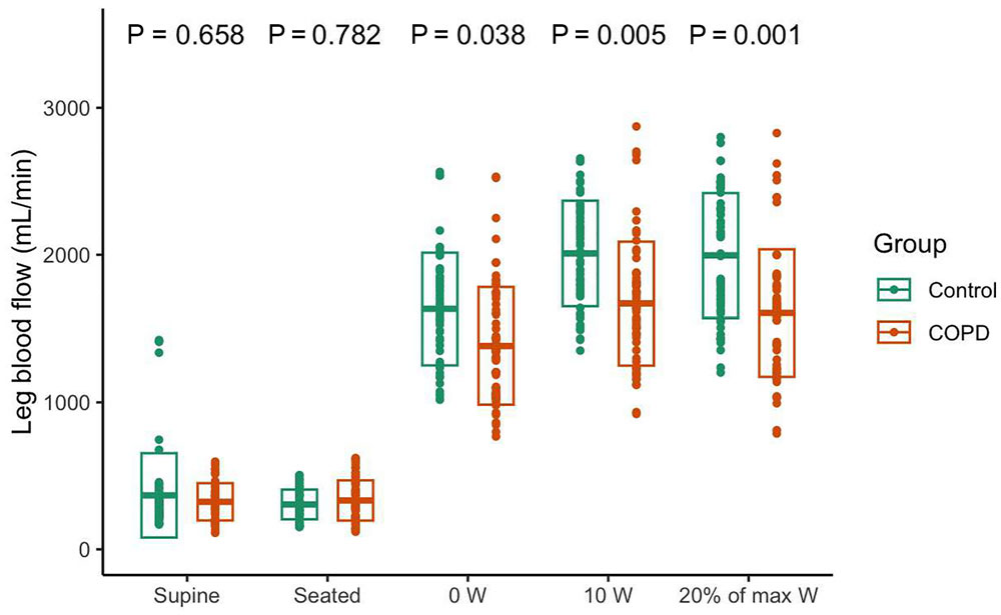
${\dot{Q}}_{\mathrm{leg}}$ of COPD and control at different intensities. ${\dot{Q}}_{\mathrm{leg}}$ in the supine and seated position, and during knee extensor exercise at 0 W, 10 W, and 20% of WL_peak_ with a dot for each measurement. Box is showing mean (SD). Red colour indicates COPD group and green colour indicates the control group. COPD, Chronic obstructive pulmonary disease; WL_peak_, maximal workload attained during the maximal knee extensor test.

For COPD patients, ${\dot{Q}}_{\mathrm{leg}}$ increased progressively across the incremental workloads (Figure 2), ranging from 323 (126) mL/min at rest to 1678 (414) mL/min during exercise at 10 W. The same was found for the healthy controls (Figure 2), ranging from 367 (286) mL/min at rest to 1974 (353) mL/min during exercise at 10 W. Both groups increased in ${\dot{Q}}_{\mathrm{leg}}$, but COPD patients had lower values compared with the healthy controls during 0 and 10 W workload, and during 20% of WL_peak_, as shown in Figure 2, with a main effect of group (*P* = 0.001).

### 3.3 Internal consistency

All data were internally consistent, as no systematic between‐day differences in ${\dot{Q}}_{\mathrm{leg}}$ were detected across intensities for either COPD or the healthy control group with no main effect of day (*P* = 0.69) (Table 2). Accordingly, the CFA diameter was the same on both days and across intensities with no main effect of day (*P* = 0.33), condition (*P* = 0.99) or group (*P* = 0.13) (Table 3). The same was applicable for CIMT with no main effect of day (*P* = 0.74), side (*P* = 0.59) or group (*P* = 0.60) (Table 4).

**TABLE 2:** Internal consistency of leg blood flow (${\dot{Q}}_{\mathrm{leg}}$) measurements.

	Day 1		Day 2	
	Measurement 1	Measurement 2	Measurement 1	Measurement 2
COPD				
Supine (mL/min)	325 (138)	314 (131)	327 (120)	328 (128)
Seated (mL/min)	323 (151)	324 (146)	337 (134)	347 (126)
0 W (mL/min)	1380 (393)	1364 (352)	1449 (429)	1461 (442)
10 W (mL/min)	1656 (380)	1624 (395)	1706 (481)	1726 (425)
20% (mL/min)	1591 (500)	1619 (415)	1637 (443)	1627 (434)
Controls				
Supine (mL/min)	351 (292)	364 (292)	380 (300)	374 (288)
Seated (mL/min)	295 (95)	313 (111)	309 (109)	304 (94)
0 W (mL/min)	1648 (385)	1655 (382)	1597 (390)	1602 (391)
10 W (mL/min)	1997 (382)	2020 (385)	1914 (333)	1964 (334)
20% (mL/min)	1982 (451)	1984 (441)	1950 (397)	1927 (376)

*Note*: Absolute ${\dot{Q}}_{\mathrm{leg}}$ values are from day 1 and day 2. The data are presented as the raw mean (SD). Abbreviations: 20%, 20% of the maximal workload attained during the maximal knee extensor test; COPD, chronic obstructive pulmonary disease.

**TABLE 3:** Internal consistency of common femoral artery (CFA) diameter measurements.

	CFA diameter (cm)	
	Day 1	Day 2
COPD		
Supine	0.98 (0.13)	0.98 (0.13)
Seated	0.98 (0.17)	0.99 (0.13)
0 W	0.98 (014)	0.99 (0.13)
10 W	0.97 (0.15)	0.99 (0.13)
20%	0.97 (0.15)	0.99 (0.13)
Controls		
Supine	1.03 (0.12)	1.04 (0.12)
Seated	1.05 (0.12)	1.06 (0.13)
0 W	1.05 (0.14)	1.06 (0.10)
10 W	1.05 (0.12)	1.07 (0.13)
20%	1.05 (0.12)	1.05 (0.12)

*Note*: Values of the common femoral artery diameter measurements were obtained on scanning day 1 and scanning day 2. The data are presented as the raw mean (SD). Abbreviations: 20%, 20% of the maximal workload attained during the maximal knee extensor test; CFA, common femoral artery; COPD, chronic obstructive pulmonary disease.

**TABLE 4:** Internal consistency of carotid intima‐media thickness (CIMT) measurements.

	Day 1		Day 2	
	Measurement 1	Measurement 2	Measurement 1	Measurement 2
COPD				
Right side (mm)	0.79 (0.17)	0.79 (0.18)	0.77 (0.17)	0.75 (0.17)
Left side (mm)	0.73 (0.12)	0.72 (0.11)	0.72 (0.11)	0.73 (0.12)
Controls				
Right side (mm)	0.71 (0.16)	0.72 (0.17)	0.70 (0.14)	0.69 (0.13)
Left side (mm)	0.72 (0.18)	0.72 (0.11)	0.76 (0.18)	0.76 (0.18)

*Note*: Values of the carotid intima‐media thickness (CIMT) are measurements obtained on scanning day 1 and scanning day 2. The data are presented as the raw mean (SD). Abbreviations: COPD, chronic obstructive pulmonary disease.

### 3.4 Between‐day test–retest reliability

For ${\dot{Q}}_{\mathrm{leg}}$, SRD is reported in Table 5 and apart from between‐group differences in supine and seated positions, it increased similarly with intensity in both groups with no additional between‐group differences (Table 5). CV was highest in both groups in the resting supine and seated position (Table 5) and decreased during exercise. CV in the resting state ranged from 18.6% to 20.4% in the COPD group and 15.8% to 17.8% in the control group. During exercise, CV ranged from 7.9% to 14.3% for the COPD group while CV ranged from 9.4% to 10.4% in the control group (Table 5). CV was higher in COPD than in controls in the seated position and at 0 W. Apart from that, no significant between‐group differences were observed. ICC varied markedly with intensity, but with no significant between‐group differences (Table 5).

**TABLE 5:** Test–retest reliability for leg blood flow (${\dot{Q}}_{\mathrm{leg}}$).

	Between‐day reliability		
	SRD (mL)	CV (%)	ICC (fraction)
COPD			
Supine	190 (149, 244)	20.4 (15.0, 26.0)	0.73 (0.52, 0.86)
Seated	178 (148, 215)	18.6 (14.0, 23.0)	0.80 (0.63, 0.90)
0 W	583 (455, 748)	14.3 (10.8, 18.0)	0.75 (0.54, 0.87)
10 W	382 (297, 491)	7.9 (6.0, 9.9)	0.90 (0.78, 0.95)
20%	367 (294, 457)	7.9 (5.9, 9.8)	0.92 (0.84, 0.96)
Control			
Supine	470 (400, 553)^a^	17.8 (13.4, 22.0)	0.95 (0.90, 0.97)
Seated	139 (108, 178)**^a^**	15.8 (11.9, 20.0)**^a^**	0.77 (0.58, 0.88)
0 W	438 (299, 642)	9.4 (7.0, 11.6)**^a^**	0.84 (0.70, 0.92)
10 W	667 (531, 838)	10.4 (7.8, 13.0)	0.67 (0.42, 0.82)
20%	542 (367, 801)	9.6 (7.0, 12.0)	0.79 (0.62, 0.89)

*Note*: Test–retest reliability measurements for leg blood flow (${\dot{Q}}_{\mathrm{leg}}$). The table presents the mean (95% CI) for between‐day reliability measurements. ^a^Different from COPD group (*P* < 0.05). Abbreviations: 20%, 20% of the maximal workload attained during the maximal knee extensor test; COPD, chronic obstructive pulmonary disease; CV, coefficient of variance; ICC, intraclass correlation coefficient; SRD, smallest real difference.

For CFA diameter, SRD, CV and ICC estimates are reported in Table 6. SRD and CV were lower in the control group both at rest and during all exercise intensities except for 20% of WL_peak_. ICC varied markedly with intensity, but with no significant between‐group differences (Table 6).

**TABLE 6:** Test–retest reliability for common femoral artery (CFA) diameter measurements.

	SRD (cm)	CV (%)	ICC (Fraction)
COPD			
Supine	0.17 (0.14, 0.21)	5.85 (4.2, 7.5)	0.84 (0.72, 0.91)
Seated	0.17 (0.13, 0.22)	5.85 (4.4, 7.3)	0.86 (0.74, 0.93)
0 W	0.14 (0.12, 0.17)	5.08 (3.8, 6.3)	0.86 (0.74, 0.93)
10 W	0.16 (0.12, 0.23)	5.55 (4.2, 6.9)	0.85 (0.71, 0.92)
20%	0.13 (0.1, 0.17)	4.42 (3.3, 5.5)	0.91 (0.83, 0.96)
Control
Supine	0.05 (0.03, 0.09)^a^	1.55 (1.2, 1.9)^a^	0.98 (0.96, 0.99)
Seated	0.14 (0.12, 0.16)^a^	4.5 (3.4, 5.6)	0.86 (0.73, 0.93)
0 W	0.11 (0.1, 0.13)^a^	3.69 (2.8, 4.6)^a^	0.90 (0.8, 0.95)
10 W	0.10^a^ (0.08, 0.12)	3.2 (2.4, 4)^a^	0.93 (0.85, 0.97)
20%	0.11 (0.08, 0.14)	3.48 (2.6, 4.3)	0.90 (0.81, 0.95)

*Note*: Test–retest reliability measurements for common femoral artery (CFA) diameter. The table presents the mean (95% CI) for between‐day reliability measurements.

^a^ Different from COPD (*P* < 0.05). Abbreviations: 20%, 20% of the maximal workload attained during the maximal knee extensor test; COPD, chronic obstructive pulmonary disease; CV, coefficient of variance; ICC, intraclass correlation coefficient; SRD, smallest real difference.

When assessing CIMT, no significant between‐group differences were found in terms of SRD, CV and ICC estimates as reported in Table 7.

**TABLE 7:** Test–retest reliability for carotid intima–media thickness (CIMT) measurements.

	SRD (cm)	CV (%)	ICC (fraction)
COPD			
Right side	0.17 (0.12, 0.24)	7.83 (5.1, 10.5)	0.86 (0.64, 0.95)
Left side	0.19 (0.11, 0.43)	8.36 (5.5, 11.2)	0.89 (0.71, 0.96)
Control			
Right side	0.15 (0.11, 0.24)	6.58 (4.3, 8.8)	0.91 (0.76, 0.97)
Left side	0.14 (0.11, 0.19)	6.59 (4.3, 8.9)	0.81 (0.53, 0.93)

*Note*: Test–retest reliability for carotid intima‐media thickness (CIMT) measurements. The table presents the mean (95% CI) for between‐day reliability measurements. Different from COPD (*P* < 0.05). Abbreviations: COPD, chronic obstructive pulmonary disease; CV, coefficient of variance; ICC, intraclass correlation coefficient; SRD, smallest real difference.

## 4 DISCUSSION

The present study is the first to estimate the test–retest reliability of Doppler ultrasound‐based ${\dot{Q}}_{\mathrm{leg}}$ measurements in patients with COPD and to compare it to age‐ and sex‐matched healthy controls. The main findings were that Doppler ultrasound provides ${\dot{Q}}_{\mathrm{leg}}$ measurements with an almost similar between‐day reliability in the two groups with the lowest CV during exercise. Moreover, we also demonstrated between‐day reliability for CFA and CIMT measurements.

The observed lower ${\dot{Q}}_{\mathrm{leg}}$ response to exercise in COPD patients compared to controls aligns with prior studies (Brønstad et al., 2012; Broxterman et al., 2021, 2020; Hartmann et al., 2022). The origin of peripheral vascular dysfunction in COPD is multifactorial. It includes altered redox balance with a reduced vascular bioavailability of nitric oxide (Ives et al., 2014) and complementary imbalance between other vasoactive substances within skeletal muscle that impairs functional sympatholysis (Haarmann et al., 2016; Ives et al., 2020; Saltin & Mortensen, 2012). Additionally, greater vascular stiffness potentially related to vascular inflammation (Eagan et al., 2010; Ives et al., 2014) in combination with reduced arterial elasticity (Kinlay et al., 2001) might also contribute to this peripheral vascular dysfunction. Consequently, this diminished ${\dot{Q}}_{\mathrm{leg}}$ may restrict exercise capacity, underscoring the importance of ${\dot{Q}}_{\mathrm{leg}}$ as a critical physiological metric in studies investigating COPD patients. In any event, the blunted vascular response to KEE in COPD aligns with theoretical predictions and previous studies, which together supports the so‐called construct validity of the measurements, that is, to what extent a measured variable change as expected in response to a given physiological perturbation and in relation to other measured variables (Hartmann et al., 2023). Given that between‐day measurements were also internally consistent at the group level, this indicates that the findings are suitable for test–retest reliability assessments.

CV was chosen as the primary relative reliability estimate because it standardises dispersion relative to the mean, allowing for easy comparison across datasets (George et al., 2000; Liu, 2012; Madsen et al., 2023; Vaz et al., 2013). ICC was selected as the secondary relative reliability measure due to its ability to assess measurement consistency. ICC reflects the proportion of total variance due to true differences between subjects, rather than random error (Koo & Li, 2016; Lee et al., 2012; Shrout & Fleiss, 1979). Previous studies on the physiolometric assessment of Doppler ultrasound‐based ${\dot{Q}}_{\mathrm{leg}}$ during KEE are scarce (Gates et al., 2009; Rådegran, 1997). Thus, no previous studies have investigated the reliability of Doppler ultrasound‐based ${\dot{Q}}_{\mathrm{leg}}$ in COPD or compared it to that of healthy age‐ and sex‐matched controls.

The present findings from both COPD patients and the control group suggest marginally superior relative reliability in terms of between‐day CV for both ${\dot{Q}}_{\mathrm{leg}}$ and CFA compared to an earlier study investigating healthy subjects (Walther et al., 2006). Moreover, our reliability data align with a study utilising Doppler ultrasound during two‐legged stepping exercise in a healthy cohort, as the study by Amin et al. (2021) showed a between‐day CV up to 15.6% during exercise, whereas the highest achieved between‐day CV during exercise in this study was 14.3% for the COPD group and even lower for the control group. Another study examining healthy participants with the same KEE set‐up as ours but in a setting not controlling factors influencing ${\dot{Q}}_{\mathrm{leg}}$ indicated a between‐day CV up to 20% at rest (Hartmann et al., 2023), similar to that of the COPD patients, but higher compared to the healthy controls from our study. This highlights the importance of a controlled setting when assessing ${\dot{Q}}_{\mathrm{leg}}$, especially if an intervention treatment is used to evaluate changes in ${\dot{Q}}_{\mathrm{leg}}$ as a physiological outcome. Altogether, this suggests that consistency in sonographer and equipment across all visits, conducted at the same time of day in the present study, may have contributed to the reliability. However, the most likely explanation is the strictly controlled setting in our study, as many factors such as fever, caffeine, alcohol, nicotine and vigorous exercise could influence ${\dot{Q}}_{\mathrm{leg}}$ and thus contribute to increased variability (Carter et al., 2011; Chaumont et al., 2018; Paterson et al., 2005; Umemura et al., 2006).

In contrast to our expectations, the between‐day reliability estimates for the COPD group were largely not different from those of the healthy matched control group. Prior studies identified the highest reliability metrics during peak ${\dot{Q}}_{\mathrm{leg}}$ (Groot et al., 2022; Lew et al., 2021), suggesting enhanced methodological dependability during exercise phases as opposed to rest periods. Notably, physiologically relevant discrepancies between groups in terms of CV and SRD were predominantly seen during the transition from rest to low‐intensity activities in between‐day reliability assessments. This phenomenon indicates that as exercise intensity escalates, the SRD also increases, reflecting the higher absolute ${\dot{Q}}_{\mathrm{leg}}$ values. This interpretation is further corroborated by the relatively stable CV, suggesting that the method's reliability is inherently robust across conditions in both groups. ICC adds very little to the above‐mentioned findings relating to CV, as ICC is sensitive to variations both within and between groups. Essentially, this implies that if ICC is reported on a highly heterogeneous population, a high within‐group standard deviation could result in a high ICC, regardless of the method's flaws (Hartmann et al., 2023).

### 4.1 Limitations

This study has some limitations that should be acknowledged. Certain variables that could potentially influence ${\dot{Q}}_{\mathrm{leg}}$, such as sleep patterns (Bain et al., 2017) and dietary intake (Johnson et al., 2011), were not regulated or accounted for. The lack of control over these factors introduces potential confounding that could influence the study outcomes.

A potential limitation of our study is the lack of group matching based on body composition. Although there were no significant differences in height, weight and BMI between the COPD and control groups, individuals with COPD exhibited a tendency towards higher body weights and increased adiposity. These factors could potentially influence the assessment of ${\dot{Q}}_{\mathrm{leg}}$, as increased adiposity may affect vascular function, accessibility and the accuracy of Doppler ultrasound measurements. Despite COPD patients tending to have higher CIMT values on average, between‐day CIMT measurements were consistent, with no significant difference in reliability estimates between the groups. Therefore, it was unlikely that CIMT served as a confounding factor for ${\dot{Q}}_{\mathrm{leg}}$ measurements.

During exercise, some individuals from the COPD group appeared to exhibit higher ${\dot{Q}}_{\mathrm{leg}}$ values compared to the control group. We speculate that this may be attributable to a few of the high individuals with COPD who had larger body mass compared to their matched control. This underscores the necessity of accounting for individual variations in body composition when interpreting data, as it complicates direct comparisons between the COPD and control group.

The COPD group in this study was on a higher number of medications, particularly long‐acting β_2_‐agonists and long‐acting muscarinic receptor antagonists, which could potentially affect the blood flow measurements. Although not directly measured, it is also speculated that the healthy control group had higher cardiopulmonary fitness than the COPD group. This difference could account for variations in the ${\dot{Q}}_{\mathrm{leg}}$ response to KEE, including higher blood flow and enhanced vascular reactivity, as well as more reliable scans due to better exercise adherence and a more accessible site for the scan. All Doppler ultrasound measurements were performed by a single sonographer. While this approach ensures consistency, it may limit the generalisability. However, most human‐experimental studies employing Doppler ultrasound during KEE do use a single sonographer, and the performance of the sonographer in the present study in terms of intra‐observer variability, i.e. test–retest reliability, is similar or even superior to that of previous studies in healthy individuals (Groot et al., 2022; Hartmann et al., 2023; Lew et al., 2021). Nevertheless, future studies should consider involving multiple sonographers to provide a more comprehensive assessment of measurement reliability across different operators and to provide interobserver variability data. Additionally, there were only two comparisons per workload/position on each study day, and this limited number of comparisons may potentially cause overestimation of reliability. Lastly, this study's cross‐sectional design limits the ability to infer causality when it comes to between‐group differences. Longitudinal studies would be particularly beneficial in understanding how ${\dot{Q}}_{\mathrm{leg}}$ dynamics evolve with disease progression and treatment interventions. Such studies could provide more definitive insights into the temporal changes in vascular function and the long‐term effects of various treatments on ${\dot{Q}}_{\mathrm{leg}}$ in COPD patients.

### 4.2 Conclusion

This study demonstrates the utility and reliability of Doppler ultrasound when assessing between‐day differences in ${\dot{Q}}_{\mathrm{leg}}$ during rest and exercise in both COPD patients and healthy controls. Despite different vascular responses between COPD patients and healthy individuals, the method proved nearly equally reliable when performed by the same sonographer in a controlled setting with a CV below 15% during KEE in both groups.

## AUTHOR CONTRIBUTIONS

Milan Mohammad: Design, data collection, data analysis, data interpretation, figures, first draft, revisions. Jacob P. Hartmann: Design, data collection, data analysis, data interpretation, figures, first draft, revisions. Amalie B. Andersen: Data collection, data interpretation, revisions. Helene L. Hartmeyer: Data collection, data interpretation, revisions. Ulrik W. Iepsen: conception, data interpretation, revisions. Ronan M. G. Berg: Conception, design, data interpretation, revisions, supervision. All authors approved the final version of the manuscript and agreed to be accountable for all aspects of the work in ensuring that questions related to the accuracy or integrity of any part of the work are appropriately investigated and resolved. Ronan Martin Griffin Berg is guarantor of this work and accepts full responsibility for the work and the conduct of the study, had access to the data, and controlled the decision to publish. All persons designated as authors qualify for authorship, and all those who qualify for authorship are listed.

## CONFLICT OF INTEREST

None of the authors have conflict of interest to declare. The authors declare that the research was conducted in the absence of any commercial or financial relationships that could be construed as a potential conflict of interest.

## Data Availability

The data underlying our findings can be shared upon reasonable request directed to the corresponding author.

## References

[eph13633-bib-0001] Agustí, A. , Celli, B. R. , Criner, G. J. , Halpin, D. , Anzueto, A. , Barnes, P. , Bourbeau, J. , Han, M. K. , Martinez, F. J. , Montes de Oca, M. , Mortimer, K. , Papi, A. , Pavord, I. , Roche, N. , Salvi, S. , Sin, D. D. , Singh, D. , Stockley, R. , López Varela, M. V. , … Vogelmeier, C. F. (2023). Global initiative for chronic obstructive lung disease 2023 report: GOLD executive summary. European Respiratory Journal, 61(4), 2300239. 36858443 10.1183/13993003.00239-2023 PMC10066569

[eph13633-bib-0002] Amin, S. B. , Mugele, H. , Dobler, F. E. , Marume, K. , Moore, J. P. , & Lawley, J. S. (2021). Intra‐rater reliability of leg blood flow during dynamic exercise using Doppler ultrasound. Physiological Reports, 9(19), e15051. 34617675 10.14814/phy2.15051 PMC8496156

[eph13633-bib-0003] Andersen, P. , & Saltin, B. (1985). Maximal perfusion of skeletal muscle in man. The Journal of Physiology, 366(1), 233–249. 4057091 10.1113/jphysiol.1985.sp015794 PMC1193029

[eph13633-bib-0004] Bain, A. R. , Weil, B. R. , Diehl, K. J. , Greiner, J. J. , Stauffer, B. L. , & DeSouza, C. A. (2017). Insufficient sleep is associated with impaired nitric oxide‐mediated endothelium‐dependent vasodilation. Atherosclerosis, 265, 41–46. 28846879 10.1016/j.atherosclerosis.2017.08.001

[eph13633-bib-0005] Barber, F. E. , Baker, D. W. , Nation, A. W. C. , Strandness, D. E. , & Reid, J. M. (1974). Ultrasonic duplex echo‐Doppler scanner. Ieee Transactions on Bio‐Medical Engineering, BME‐21(2), 109–113. 10.1109/TBME.1974.324295 4818795

[eph13633-bib-0067] Bates, D. , Mächler, M. , Bolker, B. , & Walker, S. (2015). Fitting linear mixed‐effects models usinglme4. Journal of Statistical Software, 67(1), 1–48.

[eph13633-bib-0006] Bauer, M. , Caviezel, S. , Teynor, A. , Erbel, R. , Mahabadi, A. A. , & Schmidt‐Trucksäss, A. (2012). Carotid intima‐media thickness as a biomarker of subclinical atherosclerosis. Swiss Medical Weekly, 142, w13705–w13705. 23135891 10.4414/smw.2012.13705

[eph13633-bib-0007] Bhakta, N. R. , McGowan, A. , Ramsey, K. A. , Borg, B. , Kivastik, J. , Knight, S. L. , Sylvester, K. , Burgos, F. , Swenson, E. R. , McCarthy, K. , Cooper, B. G. , García‐Río, F. , Skloot, G. , McCormack, M. , Mottram, C. , Irvin, C. G. , Steenbruggen, I. , Coates, A. L. , & Kaminsky, D. A. (2023). European Respiratory Society/American Thoracic Society technical statement: Standardisation of the measurement of lung volumes, 2023 update. European Respiratory Journal, 62(4), 2201519. 37500112 10.1183/13993003.01519-2022

[eph13633-bib-0008] Brønstad, E. , Rognmo, O. , Tjonna, A. E. , Dedichen, H. H. , Kirkeby‐Garstad, I. , Håberg, A. K. , Ingul, C. B. , Wisløff, U. , & Steinshamn, S. (2012). High‐intensity knee extensor training restores skeletal muscle function in COPD patients. European Respiratory Journal, 40(5), 1130–1136. 22408206 10.1183/09031936.00193411

[eph13633-bib-0009] Broxterman, R. M. , Hoff, J. , Wagner, P. D. , & Richardson, R. S. (2020). Determinants of the diminished exercise capacity in patients with chronic obstructive pulmonary disease: Looking beyond the lungs. The Journal of Physiology, 598(3), 599–610. 31856306 10.1113/JP279135 PMC6995414

[eph13633-bib-0010] Broxterman, R. M. , Wagner, P. D. , & Richardson, R. S. (2021). Exercise training in COPD: Muscle O2 transport plasticity. European Respiratory Journal, 58(2), 2004146. 33446612 10.1183/13993003.04146-2020

[eph13633-bib-0011] Carter, J. R. , Stream, S. F. , Durocher, J. J. , & Larson, R. A. (2011). Influence of acute alcohol ingestion on sympathetic neural responses to orthostatic stress in humans. American Journal of Physiology‐Endocrinology and Metabolism, 300(5), E771–E778. 21325108 10.1152/ajpendo.00674.2010 PMC3093974

[eph13633-bib-0012] Casella, I. B. , Presti, C. , Porta, R. M. P. , Sabbag, C. R. D. , Bosch, M. A. , & Yamazaki, Y. (2008). A practical protocol to measure common carotid artery intima‐media thickness. Clinics, 63(4), 515. 18719764 10.1590/S1807-59322008000400017 PMC2664129

[eph13633-bib-0013] Chaumont, M. , De Becker, B. , Zaher, W. , Culié, A. , Deprez, G. , Mélot, C. , Reyé, F. , Van Antwerpen, P. , Delporte, C. , Debbas, N. , Boudjeltia, K. Z. , & Van De Borne, P. (2018). Differential effects of e‐cigarette on microvascular endothelial function, arterial stiffness and oxidative stress: A randomized crossover trial. Scientific Reports 2018, 8(1), 1–9. 10.1038/s41598-018-28723-0 PMC6039507 29991814

[eph13633-bib-0014] Cornelius, T. (2024). Clinical guideline highlights for the hospitalist: GOLD COPD update 2024. Journal of Hospital Medicine, 19(9), 818–820. 38797887 10.1002/jhm.13416

[eph13633-bib-0015] Eagan, T. M. L. , Ueland, T. , Wagner, P. D. , Hardie, J. A. , Mollnes, T. E. , Damås, J. K. , Aukrust, P. , & Bakke, P. S. (2010). Systemic inflammatory markers in COPD: Results from the Bergen COPD Cohort Study. European Respiratory Journal, 35(3), 540–548. 19643942 10.1183/09031936.00088209

[eph13633-bib-0016] Gates, P. E. , Strain, W. D. , & Shore, A. C. (2009). Human endothelial function and microvascular ageing. Experimental Physiology, 94(3), 311–316. 19042980 10.1113/expphysiol.2008.043349

[eph13633-bib-0017] George, K. , Batterham, A. , & Sullivan, I. (2000). Validity in clinical research: A review of basic concepts and definitions. Physical Therapy in Sport, 1(1), 19–27.

[eph13633-bib-0018] Gill, R. W. (1985). Measurement of blood flow by ultrasound: Accuracy and sources of error. Ultrasound in Medicine & Biology, 11(4), 625–641. 2931884 10.1016/0301-5629(85)90035-3

[eph13633-bib-0019] Gliemann, L. , Mortensen, S. P. , & Hellsten, Y. (2018). Methods for the determination of skeletal muscle blood flow: Development, strengths and limitations. European Journal of Applied Physiology, 118(6), 1081–1094. 29756164 10.1007/s00421-018-3880-5

[eph13633-bib-0020] Graham, B. L. , Steenbruggen, I. , Barjaktarevic, I. Z. , Cooper, B. G. , Hall, G. L. , Hallstrand, T. S. , Kaminsky, D. A. , McCarthy, K. , McCormack, M. C. , Miller, M. R. , Oropez, C. E. , Rosenfeld, M. , Stanojevic, S. , Swanney, M. P. , & Thompson, B. R. (2019). Standardization of spirometry 2019 update an official American Thoracic Society and European Respiratory Society technical statement. American Journal of Respiratory and Critical Care Medicine, 200(8), e70–e88. 31613151 10.1164/rccm.201908-1590ST PMC6794117

[eph13633-bib-0021] Grassi, B. , Poole, D. C. , Richardson, R. S. , Knight, D. R. , Erickson, B. K. , & Wagner, P. D. (1996). Muscle O2 uptake kinetics in humans: Implications for metabolic control. Journal of Applied Physiology, 80(3), 988–998. 8964765 10.1152/jappl.1996.80.3.988

[eph13633-bib-0022] Groot, H. J. , Broxterman, R. M. , Gifford, J. R. , Garten, R. S. , Rossman, M. J. , Jarrett, C. L. , Kwon, O. S. , Hydren, J. R. , Richardson, R. S. , & Groot, C. H. J. (2022). Reliability of the passive leg movement assessment of vascular function in men. Experimental physiology, 107(5), 541–552. 35294784 10.1113/EP090312 PMC9058221

[eph13633-bib-0023] Haarmann, H. , Folle, J. , Nguyen, X. P. , Herrmann, P. , Heusser, K. , Hasenfuß, G. , Andreas, S. , & Raupach, T. (2016). Sympathetic activation is associated with exercise limitation in COPD. COPD: Journal of Chronic Obstructive Pulmonary Disease, 13(5), 589–594. 26829234 10.3109/15412555.2015.1136272

[eph13633-bib-0024] Hartmann, J. P. , Dahl, R. H. , Nymand, S. , Munch, G. W. , Ryrsø, C. K. , Pedersen, B. K. , Thaning, P. , Mortensen, S. P. , Berg, R. M. G. , & Iepsen, U. W. (2022). Regulation of the microvasculature during small muscle mass exercise in chronic obstructive pulmonary disease vs. chronic heart failure. Frontiers in Physiology, 13, 979359. 36134330 10.3389/fphys.2022.979359 PMC9483770

[eph13633-bib-0025] Hartmann, J. P. , Krabek, R. , Nymand, S. B. , Hartmeyer, H. , Gliemann, L. , Berg, R. M. G. , & Iepsen, U. W. (2023). Doppler ultrasound‐based leg blood flow assessment during single‐leg knee‐extensor exercise in an uncontrolled setting. Journal of visualized experiments: JoVE, 13(202), 10.3791/65746 38163264

[eph13633-bib-0026] Hartmann, J. P. , Olsen, M. H. , Rose, G. , Bailey, D. M. , & Berg, R. M. G. (2023). Physiolometrics and the puzzle of methodical acumen. Experimental Physiology, 108(9), 1103–1105. 37555751 10.1113/EP091406 PMC10988485

[eph13633-bib-0027] Iepsen, U. W. , Munch, G. W. , Rugbjerg, M. , Ryrsø, C. K. , Secher, N. H. , Hellsen, Y. , Lange, P. , Pedersen, B. K. , Thaning, P. , & Mortensen, S. P. (2017). Leg blood flow is impaired during small muscle mass exercise in patients with COPD. Journal of Applied Physiology, 123(3), 624–631. 28729387 10.1152/japplphysiol.00178.2017

[eph13633-bib-0028] Iepsen, U. W. , Ryrsø, C. K. , Rugbjerg, M. , Secher, N. H. , Barbosa, T. C. , Lange, P. , Thaning, P. , Pedersen, B. K. , Mortensen, S. P. , & Fadel, P. J. (2021). Cardiorespiratory responses to high‐intensity skeletal muscle metaboreflex activation in chronic obstructive pulmonary disease. Clinical Physiology and Functional Imaging, 41(2), 146–155. 33159389 10.1111/cpf.12678

[eph13633-bib-0029] Ives, S. J. , Layec, G. , Hart, C. R. , Trinity, J. D. , Gifford, J. R. , Garten, R. S. , Witman, M. A. H. , Sorensen, J. R. , & Richardson, R. S. (2020). Passive leg movement in chronic obstructive pulmonary disease: Evidence of locomotor muscle vascular dysfunction. Journal of Applied Physiology, 128(5), 1402–1411. 32324478 10.1152/japplphysiol.00568.2019 PMC7272759

[eph13633-bib-0030] Ives, S. J. , Harris, R. A. , Witman, M. A. H. , Fjeldstad, A. S. , Garten, R. S. , Mcdaniel, J. , Wray, D. W. , & Richardson, R. S. (2014). Vascular dysfunction and chronic obstructive pulmonary disease: The role of redox balance. Hypertension, 63(3), 459–467. 24324045 10.1161/HYPERTENSIONAHA.113.02255 PMC4476392

[eph13633-bib-0031] Johnson, B. D. , Padilla, J. , Harris, R. A. , & Wallace, J. P. (2011). Vascular consequences of a high‐fat meal in physically active and inactive adults. Applied Physiology, Nutrition and Metabolism, 36(3), 368–375. 10.1139/H11-028 21574775

[eph13633-bib-0032] Kinlay, S. , Creager, M. A. , Fukumoto, M. , Hikita, H. , Fang, J. C. , Selwyn, A. P. , & Ganz, P. (2001). Endothelium‐derived nitric oxide regulates arterial elasticity in human arteries in vivo. Hypertension, 38(5), 1049–1053. 11711496 10.1161/hy1101.095329

[eph13633-bib-0033] Koo, T. K. , & Li, M. Y. (2016). A guideline of selecting and reporting intraclass correlation coefficients for reliability research. Journal of Chiropractic Medicine, 15(2), 155–163. 27330520 10.1016/j.jcm.2016.02.012 PMC4913118

[eph13633-bib-0034] Köseoğlu, C. , Kurmuş, Ö. , Ertem, A. G. , Çolak, B. , Bilen, E. , İpek, G. , Durmaz, T. , Keleş, T. , & Bozkurt, E. (2016). Association between carotid intima‐media thickness and presence of coronary artery disease in chronic obstructive pulmonary disease patients. Anatolian Journal of Cardiology, 16, 601. 27004706 10.5152/AnatolJCardiol.2015.6440 PMC5368517

[eph13633-bib-0035] Lee, K. M. , Lee, J. , Chung, C. Y. , Ahn, S. , Sung, K. H. , Kim, T. W. , Lee, H. J. , & Park, M. S. (2012). Pitfalls and important issues in testing reliability using intraclass correlation coefficients in orthopaedic research. Clinics in Orthopedic Surgery, 4(2), 149–155. 22662301 10.4055/cios.2012.4.2.149 PMC3360188

[eph13633-bib-0036] Lew, L. A. , Liu, K. R. , & Pyke, K. E. (2021). Reliability of the hyperaemic response to passive leg movement in young, healthy women. Experimental Physiology, 106(9), 2013–2023. 34216162 10.1113/EP089629

[eph13633-bib-0037] Liu, S. (2012). Confidence interval estimation for coefficient of variation. Thesis. 10.57709/2785351

[eph13633-bib-0068] Madsen, A. C. , Thomsen, R. S. , Nymand, S. B. , Hartmann, J. P. , Rasmussen, I. E. , Mohammad, M. , Skovgaard, L. T. , Hanel, B. , Jønck, S. , Iepsen, U. W. , Chistensen, R. H. , Mortensen, J. , & Berg, R. M. G. (2023). Pulmonary diffusing capacity to nitric oxide and carbon monoxide during exercise and in the supine position: a test‐retest reliability study. Experimental Physiology, 108(2), 307–317. 36621806 10.1113/EP090883 PMC10103891

[eph13633-bib-0039] Maltais, F. , Decramer, M. , Casaburi, R. , Barreiro, E. , Burelle, Y. , Debigaré, R. , Dekhuijzen, P. N. , Franssen, F. , Gayan‐Ramirez, G. , Gea, J. , Gosker, H. R. , Gosselink, R. , Hayot, M. , Hussain, S. N. , Janssens, W. , Polkey, M. I. , Roca, J. , Saey, D. , Schols, A. M. , … ATS/ERS Ad Hoc Committee on Limb Muscle Dysfunction in COPD . (2014). An official American thoracic society/European respiratory society statement: Update on limb muscle dysfunction in chronic obstructive pulmonary disease. American Journal of Respiratory and Critical Care Medicine, 189(9), e15–e62. 24787074 10.1164/rccm.201402-0373ST PMC4098112

[eph13633-bib-0040] Mills, N. L. , Miller, J. J. , Anand, A. , Robinson, S. D. , Frazer, G. A. , Anderson, D. , Breen, L. , Wilkinson, I. B. , McEniery, C. M. , Donaldson, K. , Newby, D. E. , & MacNee, W. (2008). Increased arterial stiffness in patients with chronic obstructive pulmonary disease: A mechanism for increased cardiovascular risk. Thorax, 63(4), 306–311. 18024535 10.1136/thx.2007.083493

[eph13633-bib-0041] Mohamed, S. F. , Khayeka‐Wandabwa, C. , Muthuri, S. , Ngomi, N. N. , Kyobutungi, C. , & Haregu, T. N. (2023). Carotid intima media thickness (CIMT) in adults in the AWI‐Gen Nairobi site study: Profiles and predictors. Hipertensión y Riesgo Vascular, 40(1), 5–15. 36153304 10.1016/j.hipert.2022.08.001 PMC11317065

[eph13633-bib-0042] Munch, G. W. , Iepsen, U. W. , Ryrsø, C. K. , Rosenmeier, J. B. , Pedersen, B. K. , Mortensen, S. P. , Ulrik, X. , Iepsen, W. , Ryrsø, C. K. , Rosenmeier, J. B. , Pedersen, B. K. , & Mortensen, S. P. (2018). Effect of 6 wk of high‐intensity one‐legged cycling on functional sympatholysis and ATP signaling in patients with heart failure. American Journal of Physiology‐Heart and Circulatory Physiology, 314, H616–H626. 29167117 10.1152/ajpheart.00379.2017

[eph13633-bib-0043] Naqvi, T. Z. , & Lee, M. S. (2014). Carotid intima‐media thickness and plaque in cardiovascular risk assessment. JACC: Cardiovascular Imaging, 7(10), 1025–1038. 25051948 10.1016/j.jcmg.2013.11.014

[eph13633-bib-0044] Nezu, T. , Hosomi, N. , Aoki, S. , & Matsumoto, M. (2016). Carotid intima‐media thickness for atherosclerosis. Journal of Atherosclerosis and Thrombosis, 23(1), 18–31. 26460381 10.5551/jat.31989

[eph13633-bib-0045] Olsen, M. H. , Riberholt, C. R. , Berg, R. M. G. , & Møller, K. (2023). clintools: Tools for Clinical Research. *R package version 098 (CRANR‐project)* .

[eph13633-bib-0046] Paterson, N. D. , Kowalchuk, J. M. , Paterson, D. H. , & Paterson, D. H. (2005). Effects of prior heavy‐intensity exercise during single‐leg knee extension on V˙ O2 kinetics and limb blood flo. Journal of Applied Physiology, 99(4), 1462–1470. 15890756 10.1152/japplphysiol.00173.2005

[eph13633-bib-0047] Poole, D. C. , Schaffartzik, W. , Knight, D. R. , Derion, T. , Kennedy, B. , Guy, H. J. , Prediletto, R. , & Wagner, P. D. (1991). Contribution of exercising legs to the slow component of oxygen uptake kinetics in humans. Journal of Applied Physiology, 71(4), 1245–1260. 1757346 10.1152/jappl.1991.71.4.1245

[eph13633-bib-0048] Rådegran, G. (1997). Ultrasound doppler estimates of femoral artery blood flow during dynamic knee extensor exercise in humans. Journal of Applied Physiology, 83(4), 1383–1388. 9338449 10.1152/jappl.1997.83.4.1383

[eph13633-bib-0049] Rådegran, G. , & Saltin, B. (1999). Nitric oxide in the regulation of vasomotor tone in human skeletal muscle. American Journal of Physiology, 276(6), H1951–H1960. 10362675 10.1152/ajpheart.1999.276.6.H1951

[eph13633-bib-0066] Richardson, R. S. , Leek, B. T. , Gavin, T. P. , Haseler, L. J. , Mudaliar, S. R. D. , Henry, R. , Mathieu‐Costello, O. , & Wagner, P. D. (2004). Reduced mechanical efficiency in chronic obstructive pulmonary disease but normal peak V̇o2with small muscle mass exercise. American Journal of Respiratory and Critical Care Medicine, 169(1), 89–96. 14500263 10.1164/rccm.200305-627OC

[eph13633-bib-0050] Richardson, R. S. , Poole, D. C. , Knight, D. R. , Kurdak, S. S. , Hogan, M. C. , Grassi, B. , Johnson, E. C. , Kendrick, K. F. , Erickson, B. K. , & Wagner, P. D. (1993). High muscle blood flow in man: Is maximal O2 extraction compromised? Journal of Applied Physiology, 75(4), 1911–1916. 8282650 10.1152/jappl.1993.75.4.1911

[eph13633-bib-0051] Roeder, M. , Sievi, N. A. , Kohlbrenner, D. , Clarenbach, C. F. , & Kohler, M. (2020). Arterial stiffness increases over time in relation to lung diffusion capacity: a longitudinal observation study in COPD. International Journal of Chronic Obstructive Pulmonary Disease, 15, 177. 32158204 10.2147/COPD.S234882 PMC6986246

[eph13633-bib-0052] Saltin, B. , & Mortensen, S. P. (2012). Inefficient functional sympatholysis is an overlooked cause of malperfusion in contracting skeletal muscle. The Journal of Physiology, 590(24), 6269–6275. 22988143 10.1113/jphysiol.2012.241026 PMC3533189

[eph13633-bib-0053] Seto, A. H. , Tyler, J. , Suh, W. M. , Harrison, A. T. , Vera, J. A. , Zacharias, S. J. , Daly, T. S. , Sparling, J. M. , Patel, P. M. , Kern, M. J. , & Abu‐Fadel, M. (2017). Defining the common femoral artery: Insights from the femoral arterial access with ultrasound trial. Catheterization and Cardiovascular Interventions, 89(7), 1185–1192. 27566991 10.1002/ccd.26727

[eph13633-bib-0054] Shrout, P. E. , & Fleiss, J. L. (1979). Intraclass correlations: Uses in assessing rater reliability. Psychological Bulletin, 86(2), 420–428. 18839484 10.1037//0033-2909.86.2.420

[eph13633-bib-0055] Stanojevic, S. , Kaminsky, D. A. , Miller, M. R. , Thompson, B. , Aliverti, A. , Barjaktarevic, I. , Cooper, B. G. , Culver, B. , Derom, E. , Hall, G. L. , Hallstrand, T. S. , Leuppi, J. D. , MacIntyre, N. , McCormack, M. , Rosenfeld, M. , & Swenson, E. R. (2022). ERS/ATS technical standard on interpretive strategies for routine lung function tests. European Respiratory Journal, 60(1), 2101499. 34949706 10.1183/13993003.01499-2021

[eph13633-bib-0056] Umemura, T. , Ueda, K. , Nishioka, K. , Hidaka, T. , Takemoto, H. , Nakamura, S. , Jitsuiki, D. , Soga, J. , Goto, C. , Chayama, K. , Yoshizumi, M. , & Higashi, Y. (2006). Effects of acute administration of caffeine on vascular functio*n* . The American journal of cardiology, 98(11), 1538–1541. 17126666 10.1016/j.amjcard.2006.06.058

[eph13633-bib-0057] van den Oord, S. C. H. , Sijbrands, E. J. G. , ten Kate, G. L. , van Klaveren, D. , van Domburg, R. T. , van der Steen, A. F. W. , & Schinkel, A. F. L. (2013). Carotid intima‐media thickness for cardiovascular risk assessment: Systematic review and meta‐analysis. Atherosclerosis, 228(1), 1–11. 23395523 10.1016/j.atherosclerosis.2013.01.025

[eph13633-bib-0058] Vaz, S. , Falkmer, T. , Passmore, A. E. , Parsons, R. , & Andreou, P. (2013). The case for using the repeatability coefficient when calculating test–retest reliability. PLoS ONE, 8(9), e73990. 24040139 10.1371/journal.pone.0073990 PMC3767825

[eph13633-bib-0059] von Elm, E. , Altman, D. G. , Egger, M. , Pocock, S. J. , Gøtzsche, P. C. , & Vandenbroucke, J. P. (2008). The Strengthening the Reporting of Observational Studies in Epidemiology (STROBE) statement: Guidelines for reporting observational studies. Journal of Clinical Epidemiology, 61(4), 344–349. 18313558 10.1016/j.jclinepi.2007.11.008

[eph13633-bib-0060] Walløe, L. , & Wesche, J. (1988). Time course and magnitude of blood flow changes in the human quadriceps muscles during and following rhythmic exercise. The Journal of Physiology, 405(1), 257–273. 3255792 10.1113/jphysiol.1988.sp017332 PMC1190975

[eph13633-bib-0061] Walther, G. , Nottin, S. , Dauzat, M. , & Obert, P. (2006). Femoral and axillary ultrasound blood flow during exercise: A methodological study. Medicine and Science in Sports and Exercise, 38(7), 1353–1361. 16826035 10.1249/01.mss.0000227323.69588.f4

[eph13633-bib-0062] Wesche, J. (1986). The time course and magnitude of blood flow changes in the human quadriceps muscles following isometric contraction. The Journal of Physiology, 377(1), 445–462. 3795098 10.1113/jphysiol.1986.sp016197 PMC1182843

[eph13633-bib-0063] Willeit, P. , Tschiderer, L. , Allara, E. , Reuber, K. , Seekircher, L. , Gao, L. , Liao, X. , Lonn, E. , Gerstein, H. C. , Yusuf, S. , Brouwers, F. P. , Asselbergs, F. W. , van Gilst, W. , Anderssen, S. A. , Grobbee, D. E. , Kastelein, J. J. P. , Visseren, F. L. J. , Ntaios, G. , Hatzitolios, A. I. , … PROG‐IMT and the Proof‐ATHERO Study Groups . (2020). Carotid intima‐media thickness progression as surrogate marker for cardiovascular risk: Meta‐Analysis of 119 Clinical Trials Involving 100 667 Patients. Circulation, 142(7), 621–642. 32546049 10.1161/CIRCULATIONAHA.120.046361 PMC7115957

[eph13633-bib-0064] Zhang, X. H. , Zhang, S. T. , Huang, Q. L. , Liu, Y. Q. , Chang, J. N. , & Liu, P. (2022). Comparison of arterial stiffness and ultrasound indices in patients with and without chronic obstructive pulmonary disease. Revista Da Associacao Medica Brasileira, 68(5), 605–609. 35584482 10.1590/1806-9282.2021203

